# Composition and Dynamics of Bacterial Communities in a Full-Scale Mineral Water Treatment Plant

**DOI:** 10.3389/fmicb.2019.01542

**Published:** 2019-07-24

**Authors:** Lei Wei, Qingping Wu, Jumei Zhang, Weipeng Guo, Qihui Gu, Huiqing Wu, Juan Wang, Tao Lei, Moutong Chen, Musheng Wu, Aimei Li

**Affiliations:** ^1^School of Bioscience and Bioengineering, South China University of Technology, Guangzhou, China; ^2^State Key Laboratory of Applied Microbiology Southern China, Guangdong Provincial Key Laboratory of Microbial Culture Collection and Application, Guangdong Open Laboratory of Applied Microbiology, Guangdong Institute of Microbiology, Guangdong Academy of Science, Guangzhou, China; ^3^College of Food Science, South China Agricultural University, Guangzhou, China; ^4^Guangdong Dinghu Mountain Spring Company Limited, Zhaoqing, China

**Keywords:** mineral water treatment system, bacterial community, high-throughput sequencing, core microbiome, opportunistic pathogens

## Abstract

The aim of this study was to gain insight into the bacterial composition and dynamics in a mineral water treatment system (MWTS). The bacterial community of a full-scale mineral water treatment plant in the Maofeng Mountain, South China, was studied using high-throughput sequencing combined with cultivation-based techniques in both the dry and wet season. Overall, adenosine tri-phosphate (ATP) concentration (6.47 × 10^-11^ – 3.32 × 10^-8^ M) and heterotrophic plate counts (HPC) (3 – 1.29 × 10^3^ CFU/mL) of water samples in the wet season were lower than those (ATP concentration 5.10 × 10^-11^ – 6.96 × 10^-8^ M, HPC 2 – 1.97 × 10^3^ CFU/mL) in the dry season throughout the whole MWTS. The microbial activity and biomass of water samples obviously changed along with treatment process. All 300 isolates obtained using cultivation-based techniques were distributed in 5 phyla, 7 classes, and 19 genera. *Proteobacteria* accounted for 55.7% (167) of the total isolates, among which predominant genus was *Pseudomonas* (19.3%). Illumina sequencing analysis of 16s rRNA genes revealed 15 bacterial phyla (relative abundance >0.1%) as being identified in all water samples. Among these, *Proteobacteria* constituted the dominant bacteria microbiota in all water samples. A large shift in the proportion of *Bacteroidetes, Actinobacteria*, and *Firmicutes* was obtained during the treatment process, with the proportion of *Bacteroidetes, Actinobacteria* decreasing sharply, whereas that of *Firmicutes* increased and predominated in the final water product. The core microbiome, which was still present in whole MWTS comprised several genera including *Pseudomonas*, *Acinetobacter*, *Clostridium*, and *Mycobacterium*, that contain species that are opportunistic pathogens, suggesting a potential threat for mineral water microbiology safety. This study is the first to investigate the bacterial community of a full-scale mineral water treatment plant in China. The results provided data regarding the bacteria composition and dynamics in an MWTS, which will contribute to the beneficial manipulation of the mineral water microbiome.

## Introduction

Large-scale outbreaks of infectious diseases via waterborne pathogens constitute a public serious health problem (Faria et al., 2009; Liu et al., 2018). It is cautiously estimated that approximately 350 million people worldwide are infected with waterborne pathogens and 25 million children annually die from diseases caused by drinking contaminated water (Eichler et al., 2006; Deere et al., 2017). Although numerous strictly regulated microbial indicators exist for mineral water, the production of mineral water that meets quality standards does not necessarily guarantee microbial safety (Varga, 2011; Zhang et al., 2015). In particular, several recent studies have found that mineral water contains pathogens and opportunistic pathogens, which can adversely affect the health of consumers (Herath et al., 2012; Wei et al., 2017). The bacterial community is ubiquitous throughout the entire mineral water treatment system (MWTS) (Bharath et al., 2003; Pontara et al., 2011). Therefore, a comprehensive understanding of the composition and dynamics of bacterial communities in the MWTS is essential to guarantee the microbial safety of mineral water.

The spatiotemporal heterogeneity of microbial communities in drinking water treatment system (DWTS) has been investigated in several previous studies. For example, Pinto et al. (2014) found that the microbial community structure exhibits a large temporal change in DWTS. However, some studies have also shown that the primary dynamic pattern of variations in bacterial communities occur during the treatment process rather than via temporal fluctuations (El-Chakhtoura et al., 2015; Li et al., 2017). Hou et al. (2018) reported that microbial community structure had temporal change in DWTS and the treatment processes exerted profound influence on the microbial community composition. However, compared to DWTS, little information is available regarding the spatiotemporal variations of multi-step treatment processes in MWTS, which cannot guarantee the microbial safety of the final mineral water product.

The production process of mineral water mainly includes three levels of filter (quartz sand, activated carbon, and fine filter), ozone sterilization, filling and capping, and light inspection of the finished product (Zhang et al., 2011; Wei et al., 2017). Although some studies have focused on the raw water and the final water product in MWTS, the information is still lacking regarding on the spatiotemporal changes of the bacterial communities in the whole treatment process. Moreover, how each treatment process and season affects the bacterial community structure of mineral water remains unclear, and it is unknown whether common microbial patterns exist in the MWTS. This information was very important for ensuring the microbial safety of mineral water production. In addition, most studies regarding the bacterial communities have been conducted using a single method, such as cultivation-based techniques or molecular biological methods. However, as 90% of microorganisms are uncultured, the results of cultivation-based techniques cannot accurately reflect the actual information of bacterial communities (Griebler and Lueders, 2009; Albertsen et al., 2013). Molecular biology technology which avoids the technical difficulty of obtaining uncultured microorganisms, has fundamental disadvantages. Molecular biology technology does not allow an accurate understanding of the physiological and metabolic characteristics of microorganisms, which is unable to be effectively applied for the control and utilization of microorganisms (Hahn et al., 2016; Pulschen et al., 2017). Therefore, the actual composition and characteristics of the mineral water microbial community needs to be explored in more detail through the use of high-throughput sequencing analysis combined with cultivation techniques.

To our knowledge, a systematic survey of the bacterial community in MWTS has not previously been conducted in China. In the present study, the bacterial community of a full-scale mineral water treatment plant in the Maofeng Mountain, South China was studied using Illumina HiSeq sequencing analyses combined with cultivation-based techniques in both dry and wet season. The aims of this study were to understand the composition and dynamics of the bacterial community in whole MWTS and investigate whether potential pathogens and opportunistic pathogens exist across the mineral water. The information generated in this study will provide strong basis for ensuring the microbial safety of mineral water.

## Materials and Methods

### Study Site and Sample Collection

**FIGURE 1:**
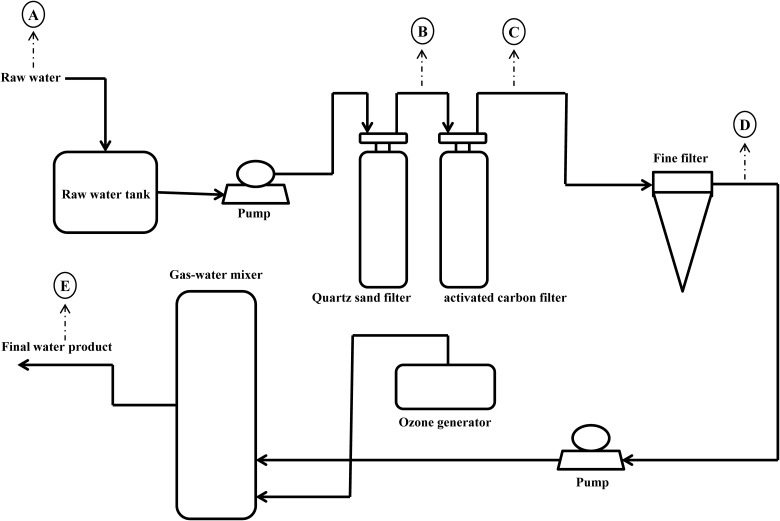
Production flow chart of mineral water in China.

The mineral water treatment plant is located in the Maofeng Mountain, south China. Its raw water originates from the groundwater at a depth of 200 meter. The production process of mineral water is shown in Figure 1. Water samples were collected in sterile containers from raw water (A), quartz sand filtered water (B), activated carbon filtered water (C), fine filtered water (D), and final water product (E) in the wet season and dry season in 2017. For each sampling point, three replicate samples were collected. All water samples were maintained at 4°C during transportation and were transported to laboratory within 4 h after sample collection. After all the samples arrived in the laboratory, 2 L of raw water, 5 L of quartz sand filtered water, 5 L of activated carbon filtered water, 10 L of fine filtered water and 10 L of final water product were filtered through sterile 0.22 μm pore-sized polycarbonate membranes (Millipore, Billerica, MA, United States). Membranes were transferred to sterile tubes and stored at -80°C. Each water sample was retested three times. The remainders of the sample were stored at 4°C, until all assayed parameters were confirmed.

### Water Quality Analysis

To understand the water quality of all water samples, several standard physicochemical parameters were measured. Turbidity was measured using a turbidimeter (Mettler Toledo, Zurich, Switzerland). Temperature and pH was measured *in situ* using a multi-parameter water quality monitoring sonde (INESA Scientific, Shanghai, China). Other physicochemical parameters, including ammonia nitrogen, chemical oxygen demand (COD), total organic carbon (TOC) were measured according to standard examination methods for drinking water (GB/T 5750-2006, China). Ammonia nitrogen was determined by spectrophotometry. COD was measured by titration. TOC was measured using a total machine carbon analyzer (Shimadzu, Kyoto, Japan). All determinations were performed in triplicate. As groundwater was confined water and the dissolved oxygen content is very low, the parameter of dissolved oxygen (DO) was not measured.

### Microbial Biomass and Activity

Heterotrophic plate counts (HPC) of all water samples were determined using a spread plating method on Reasoner’s 2A (R2A) agar (Haibo Co., Qingdao, China) (Lehtola et al., 2004). Briefly, all water samples were diluted to the appropriate concentration and 0.2 mL diluent was spread on R2A agar plate. The plates were cultured at 20°C for 7 days before colony-forming units (CFU) counting. The microbial activity was characterized by measuring adenosine tri-phosphate (ATP). ATP concentration was detected using a bioluminescence assay kit (Huankai Co., Guangzhou, China) described by Zhang et al. (2011). Briefly, membranes that filter water samples were fully washed with 0.5 mL ddH_2_O, and were reacted with 0.5 mL ATP-releasing reagent for 2 min. 0.1 mL reaction solution, 0.2 mL buffer solution, 0.1 mL luminescent reagent were added into a cuvette in sequence and luminous pulse value was detected by Glomax 20/20 luminometer (Promega Co., Madison, WI, United States). The ATP concentration of each sample was calculated according to a calibration curve generated using standards of known ATP. All determinations were performed in triplicate.

### Isolation and Identification of Bacteria From Raw Water

Three kinds of medium, nutrient agar (NA) (Huankai Co., Guangzhou, China), trypticase soy agar (TSA) (Huankai Co., Guangzhou, China), and R2A (Haibo Co., Qingdao, China) were used as separate medium for the isolation of bacteria from raw water. Following incubation in 20°C culture for 7 days, colonies with different morphologies (color, size, edge, and transparency) were purified using the streaking method. After purification, all the isolates were cultured with R2A liquid medium to preserve and extract the genomic DNA. The genomic DNA of the isolate was extracted according to the specifications of the Bacterial Genomic DNA Purification Kit (Dongsheng Biotech, Guangzhou, China). Primers 27F (5′-GTGCTGCAGAGAGTTTGATCCTGGCTCAG-3′) and 1492R (5′-CACGGATCCTACGGGTACCTTGTTACGACTT-3′) were used to amplify the 16S rRNA gene of each isolate (Dymock et al., 1996). The polymerase chain reaction (PCR) mixture contained 2 μL of template, 12.5 μL PCR Taq-mix, 0.5 μL of each primer, and 8.5 μL of ddH_2_O. Amplifications were performed using a DNA thermocycler under the following temperature profiles: 5 min of denaturation at 95°C and 35 cycles of 30 s at 95°C, 45 s at 56°C, and 1.5 min at 72°C, followed by an additional 10 min at 72°C at the end of the 35th cycle for repair and extension. The PCR products were electrophoresed using 1.0% (W/V) agarose gel, and the positive PCR products were selected and sent to Huada Gene Co., (Guangzhou, China) for sequencing using the PCR primers. According to a previously described method (Kim et al., 2012), 16S rDNA sequences were divided into different Operational Taxonomic Units (OTUs) and representative 16S rDNA sequences were selected in each OTU. Representative 16S rDNA sequences were submitted to the National Center for Biotechnology Information (NCBI) and GenBank databases for comparison and identification using BLAST software.

### Microbial Community Analysis

The polycarbonate membranes that had been used to analyze the microbial community were cut into pieces with sterilized scissors. Total genomic DNA of all the samples was extracted according to the specifications of the Powersoil DNA extraction kit (Mobio Laboratories, Carlsbad, CA, United States). PCR amplicon libraries were constructed for Illumina HiSeq sequencing using bacterial primers 341F (5′- CCTAYGGGRBGCASCAG-3′) and 806R (5′-GGACTACHVGGGTWTCTAAT-3′) targeting the V3+V4 hypervariable regions of the 16S rRNA genes (Wu et al., 2015). The PCR mixture (20 μL) contained 10 ng of template DNA, 4 mL of 5× FastPfu Buffer, 2.5 mM dNTPs, 0.4 mL FastPfu Polymerase, 5 M of each primer, and ddH_2_O. Amplifications were performed as follows: an initial denaturation at 95°C for 5 min; followed by 27 cycles at 95°C for 30 s, 55°C for 30 s, and 72°C for 45 s; and a final extension at 72°C for 10 min. The obtained PCR products were purified and joined using a sequencing linker. After constructing the gene library, the modified products were subjected to high-throughput sequencing using Illumina Hiseq2500 (San Diego, CA, United States). The reads from the original DNA fragments were merged using FLASH^1^ (Magoč and Salzberg, 2011). Quality filtering was carried out on the initial data obtained by sequencing to obtain more accurate and high-quality DNA sequence. To obtain high-quality sequences (primer mismatch base number <1%, sequence length 200–500 bp, no chimeras), the original sequences were processed using the protocol described by Caporaso et al. (2010). Sequences were grouped into OTUs at 97% sequence similarity using Mothur version 1.34.0^2^. To obtain the species classification information of each OTU, the representative sequences for each OTU were selected and submitted for taxonomic identification using the Ribosomal Database Project (RDP) classifier (see text footnote 2) (Wang et al., 2007). Mothur version 1.34.0 was used as the processing pipeline for calculation of alpha-diversity indices including the Chao1, Simpson, and Shannon indices. Based on the relative abundance of bacterial phyla, unweighted UniFrac with QIIME^3^ was used for the unweighted pair-group method with arithmetic mean clustering. Principal component analysis (PCA) and canonical correspondence analysis (CCA) were conducted using Canoco 4.5 software^4^.

### Data Analysis

All the data were processed using Microsoft Excel 2010 software (Redmond, WA, United States) and statistical analysis was performed using IBM SPSSV20.0 software (Armonk, NY, United States). The differences between groups were analyzed using one-way analysis of variance (ANOVA) and the statistical significance level was set at *p* = 0.05.

## Results

### Water Quality

As shown in Table 1, a series of specific physicochemical parameters were determined for all water samples. The raw water temperature in the wet season and the dry season were approximately 20.0°C and 20.9°C, respectively, and has no obvious change in the follow-up process treatment. The pH of all the water samples was stable in the range of 7.3 to 7.5 across the whole MWTS in both seasons. The concentrations of ammonia in whole MWTS were <0.02 mg/L in both seasons. The turbidity, TOC, and COD_Mn_ of raw water samples were 0.60 nephelometric turbidity units (NTU), 0.61 and 0.41 mg/L in the wet season, and 0.70 NTU, 0.74, and 0.45 mg/L in the dry season, respectively. As the seasons changed, the turbidity, TOC, and COD_Mn_ of the water samples showed no significant difference in whole MWTS (*p* > 0.05). However, the turbidity, TOC, and COD_Mn_ of the water samples were changed by most treatment processes (*p* < 0.05).

**Table 1:** Physicochemical parameters of 5 water samples in whole MWTS.

Samples	Temperature (°C)	pH	Turbidity (NTU)	TOC (mg/L)	COD_Mn_ (mg/L)	Ammonia-N (mg/L)
**Samples in the wet season**					
A	20.0 ± 0.3	7.5 ± 0.1	0.60 ± 0.04	0.61 ± 0.08	0.41 ± 0.02	<0.02
B	19.6 ± 0.3	7.4 ± 0.1	0.38 ± 0.04^##^	0.47 ± 0.03^##^	0.34 ± 0.02^##^	<0.02
C	19.0 ± 0.2	7.4 ± 0	0.09 ± 0.02^##^	0.57 ± 0.01^#^	0.35 ± 0.04	<0.02
D	19.4 ± 0.1	7.3 ± 0.1	0.05 ± 0.01	0.37 ± 0.05^##^	0.18 ± 0.04^##^	<0.02
E	19.7 ± 0.4	7.3 ± 0.1	0.02 ± 0.01	0.36 ± 0.05	0.17 ± 0.01	<0.02
**Samples in the dry season**					
A	20.9 ± 0.1	7.5 ± 0	0.70 ± 0.05^∗∗^	0.74 ± 0.03^∗∗^	0.45 ± 0.02	<0.02
B	20.6 ± 0.3	7.5 ± 0.1	0.41 ± 0.03^##^	0.46 ± 0.07^##^	0.34 ± 0.01^##^	<0.02
C	20.8 ± 0.2	7.5 ± 0.1	0.11 ± 0.03^##^	0.58 ± 0.01^##^	0.31 ± 0.02	<0.02
D	20.7 ± 0.1	7.4 ± 0.1	0.05 ± 0.01^#^	0.38 ± 0.05^##^	0.18 ± 0.06^##^	<0.02
E	20.7 ± 0.1	7.3 ± 0.1	0.02 ± 0.01	0.39 ± 0.03	0.20 ± 0.04	<0.02


*A, represents raw water; B, represents quartz sand filtered water; C, represents activated carbon filtered water; D, represents fine filtered water; E, represents final water product. ^∗∗^represents sample in dry season significantly different from sample in wet season (*p* < 0.01); #represents the sample significantly different from its front sample (*p* < 0.05); ^##^represents the sample significantly different from its front sample (*p* < 0.01).*

### Microbial Biomass and Microbial Activity

**FIGURE 2:**
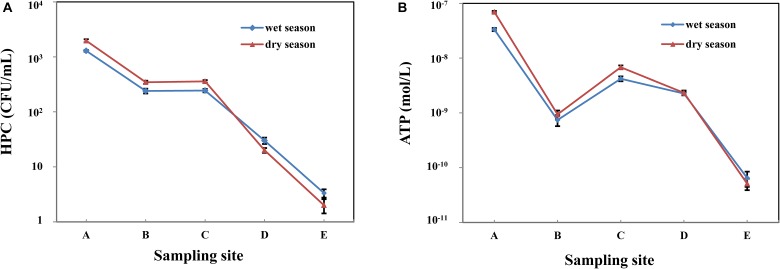
Variations of heterotrophic plate counts (HPC) **(A)** and bacterial adenosine tri-phosphate (ATP) concentrations **(B)** in whole MWTS. A, represents raw water; B, represents quartz sand filtered water; C, represents activated carbon filtered water; D, represents fine filtered water; E, represents final water product.

The HPC from raw water in the wet and dry season was 1.29 × 10^3^ and 1.97 × 10^3^ CFU/mL, respectively (Table 2). Along with the treatment process in whole MWTS, the HPC showed a downward trend in both seasons (Figure 2A). After filtration by quartz sand and fine filter, the microbial biomass decreased sharply (*p* < 0.01). However, after activated carbon filtration, the HPC showed no significant change. The HPC of all finished water in both seasons was <4 CFU/mL. The microbial activity of water samples in the wet season was lower than that in the dry season in whole MWTS, especially in the raw water and activated carbon filtered water (*p* < 0.01). ATP concentration during the treatment process ranged from 3.32 × 10^-8^ to 6.47 × 10^-11^ M in the wet season and 6.96 × 10^-8^ to 5.10 × 10^-11^ M in the dry season (Figure 2B). Along with treatment process, the ATP concentration was obviously reduced after quartz sand filter, and then exhibited a significant increase after activated carbon filter in both season (*p* < 0.01). The ATP concentration of activated carbon filtered water was 4.20 × 10^-9^ M in the wet season and 6.81 × 10^-9^ M in the dry season.

**Table 2:** Heterotrophic plate counts (HPC) and bacterial adenosine tri-phosphate (ATP) concentrations of 5 water samples in whole MWTS.

Samples	HPC (CFU/mL)	ATP (× 10^-12^ mol/L)
**Samples in the wet season**		
A	1287.7 ± 50.3	33245.7 ± 2592.4
B	240.0 ± 24.0^##^	740.0 ± 168.2^##^
C	244.0 ± 17.8	4203.3 ± 261.9^##^
D	30.0 ± 4.0^##^	2258.3 ± 297.7^#^
E	3.3 ± 0.6	64.7 ± 20.0^#^
**Samples in the dry season**		
A	1966.7 ± 130.1^∗∗^	69620.0 ± 1624.3^∗∗^
B	346.0 ± 22.5*##	937.3 ± 67.0^##^
C	359.3 ± 17.9^∗∗^	6810.3 ± 384.6^∗∗##^
D	20.0 ± 2.0^##^	2324.3 ± 237.4^##^
E	2.3 ± 0.6	51.0 ± 12.2^##^


*A, represents raw water; B, represents quartz sand filtered water; C, represents activated carbon filtered water; D, represents fine filtered water; E, represents final water product. ^∗^represents sample in dry season significantly different from sample in wet season (*p* < 0.05); ^∗∗^represents sample in dry season significantly different from sample in wet season (*p* < 0.01); ^#^represents the sample significantly different from its front sample (*p* < 0.05); ^##^represents the sample significantly different from its front sample (*p* < 0.01).*

### Isolation and Identification of Bacteria From Groundwater

In total, 141 isolates from the wet season and 159 isolates from the dry season were isolated in raw water samples (Supplementary Table S1). A total of 139 isolates were cultured from R2A medium, 93 isolates from NA medium, and 68 isolates from TSA medium. According to 16s rRNA gene sequence, all the isolates could be divided into 23 OTUs (Table 3). All 300 isolates were distributed in 5 phyla (*Proteobacteria*, *Chloroflexi*, *Firmicutes*, *Bacteroides*, and *Actinobacteria*), 7 classes (*α-proteobacteria*, *β-proteobacteria*, *γ-proteobacteria*, *Chloroflexi*, *Bacilli*, *Flavobacteriia*, and *Actinomycete*) and 19 genera (*Pseudomonas*, *Chryseobacterium*, *Porphyrobacter*, *Xanthomonas*, *Brevundimonas*, *Caulobacter*, *Acinetobacter*, *Sphingomonas*, *Bacillus*, *Brevibacterium*, *Arthrobacter*, *Aquimonas*, *Chloroflexus*, *Mycobacterium*, *Roseomonas*, *Chromobacterium*, *Pimelobacter*, *Microbacterium*, and *Hydrogenophaga*). *Proteobacteria* accounted for 55.7% (167) of the total isolates, followed by *Actinobacteria* accounting for 23.7% (71). Among all the isolates, the predominant genera were *Pseudomonas* (19.3%) and *Sphingomonas* (7.7%) at genus level. All the isolates cultured from R2A medium were distributed in 18 different genera, and *Porphyrobacter*, *Bacillus*, *Chromobacterium*, *Roseomonas*, and *Pimelobacter* were isolated only on R2A medium. *Mycobacterium* and *Pimelobacter* were isolated only from the sample in the wet season.

**Table 3:** Blast results and classify information of representative isolates cultured from raw water sample.

OTU No.	Sequence No.	Representative Strain No.	Genus
OTU 1	39	SC14	*Pseudomonas*
OUT 2	8	NFKQ49	*Chryseobacterium*
OTU 3	11	SC39	*Porphyrobacter*
OTU 4	6	CSC23	*Xanthomonas*
OTU 5	20	CSC34	*Brevundimonas*
OTU 6	16	BM24	*Caulobacter*
OTU 7	8	sbb36	*Acinetobacter*
OTU 8	23	SC32	*Sphingomonas*
OTU 9	14	sbb53	*Bacillus*
OTU 10	19	GZJT26	*Pseudomonas*
OTU 11	3	sbb29	*Acinetobacter*
OTU 12	20	sbb13	*Brevibacterium*
OTU 13	10	sbb50	*Arthrobacter*
OTU 14	4	BM14	*Xanthomonas*
OTU 15	6	CSC12	*Chryseobacterium*
OTU 16	14	NFKQ9	*Aquimonas*
OTU 17	24	sbb39	*Chloroflexus*
OTU 18	6	CSC40	*Mycobacterium*
OTU 19	7	SC42	*Roseomonas*
OTU 20	14	CSC36	*Chromobacterium*
OTU 21	6	NFKQ39	*Pimelobacter*
OTU 22	18	sbb51	*Microbacterium*
OTU 23	4	BM43	*Hydrogenophaga*


*The operational taxonomic units (OTUs) were defined with 97% similarity.*

### Diversity of Bacterial Community

**FIGURE 3:**
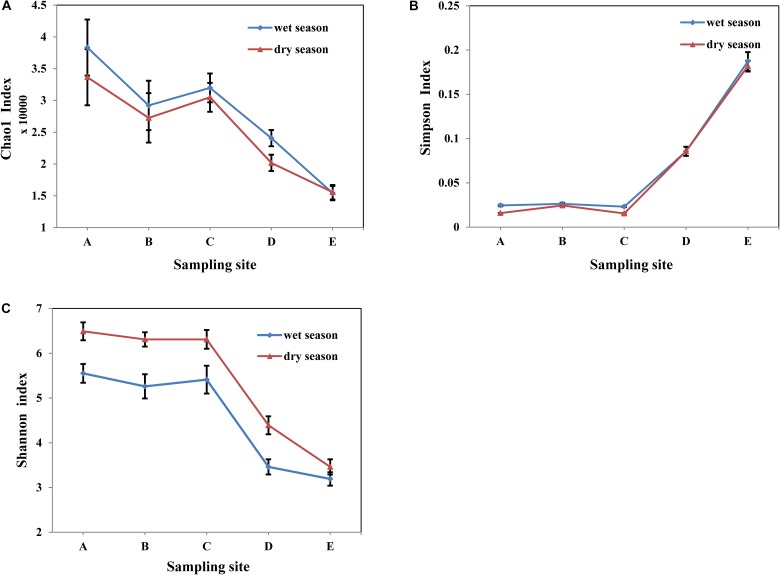
Variations of Diversity indices, Chao1 index **(A)**, Simpson index **(B)**, and Shannon index **(C)** in whole MWTS. A, represents raw water; B, represents quartz sand filtered water; C, represents activated carbon filtered water; D, represents fine filtered water; E, represents final water product.

A range of 20,693 to 39,643 original bacterial 16S rRNA gene sequences were extracted from each sample using Illumina HiSeq sequencing analysis (Table 4). The OTUs of the water samples in the wet season ranged from 2411 to 6607, and ranged 2524 to 7391 for sample in the dry season. As shown in Figure 3, In general, the diversity indices of water samples in the dry season were comparable to those in the wet season. Among all the water samples in both seasons, the diversity indices of raw water samples were the highest. Chao1 richness index ranged from 15421 to 38316 in the wet season, and ranged 15567 to 33647 in the dry season. Shannon index ranged from 3.19 to 5.55 in the wet season, and ranged 3.46 to 6.49 in the dry season. Along with treatment process in whole MWTS, the richness index of Chao1 and the Shannon index gradually decreased to the lowest level and were significantly changed by the fine filtration and ozone sterilization treatments. Simpson diversity index of finished water was 0.1872 in the wet season and 0.1819 in the dry season. However, those of raw water reached 0.0245 and 0.0158 in the wet and dry seasons, respectively.

**Table 4:** Bacterial community richness and diversity indices for 5 water samples in whole MWTS.

Sample	Reads	OTUs^a^	Chao1	Simpson	Shannon
**Samples in the wet season**					
A	39430 ± 3287	6349 ± 634	38316 ± 9440	0.0245 ± 0.0009	5.55 ± 0.20
B	32651 ± 4043^##^	6607 ± 204	29218 ± 3887^##^	0.0263 ± 0.0012	5.26 ± 0.16
C	39229 ± 3780	6297 ± 40	31977 ± 2270	0.0231 ± 0.0005	5.41 ± 0.21
D	27782 ± 2219^##^	4428 ± 217^##^	24072 ± 1277^#^	0.0856 ± 0.0052^##^	3.46 ± 0.20^##^
E	21511 ± 1528^#^	2411 ± 108^##^	15421 ± 1135^##^	0.1872 ± 0.0105^##^	3.19 ± 0.17
**Samples in the dry season**					
A	39643 ± 2783	7120 ± 336^∗∗^	33647 ± 2147	0.0158 ± 0.0007*	6.49 ± 0.21^∗∗^
B	35601 ± 2102	7391 ± 76^∗∗^	27259 ± 853^#^	0.0244 ± 0.0010^#^	6.31 ± 0.27^∗∗^
C	38927 ± 2211	7253 ± 260^∗∗^	30495 ± 2141	0.0154 ± 0.0010^#^	6.31 ± 0.31^∗∗^
D	26267 ± 2181^##^	4751 ± 163^##^	20179 ± 2461^#^	0.0858 ± 0.0047^##^	4.39 ± 0.17^∗∗##^
E	20693 ± 1526^##^	2524 ± 214^##^	15567 ± 2117	0.1819 ± 0.0067^##^	3.46 ± 0.15^##^


*A, represents raw water; B, represents quartz sand filtered water; C, represents activated carbon filtered water; D, represents fine filtered water; E, represents final water product; ^∗^represents sample in dry season significantly different from sample in wet season (*p* < 0.05); ^∗∗^represents sample in dry season significantly different from sample in wet season (*p* < 0.01); ^#^represents the sample significantly different from its front sample (*p* < 0.05); ^##^represents the sample significantly different from its front sample (*p* < 0.01). ^*a*^represents the operational taxonomic units (OTUs) were defined with 97% similarity.*

### Composition of Bacterial Community

**FIGURE 4:**
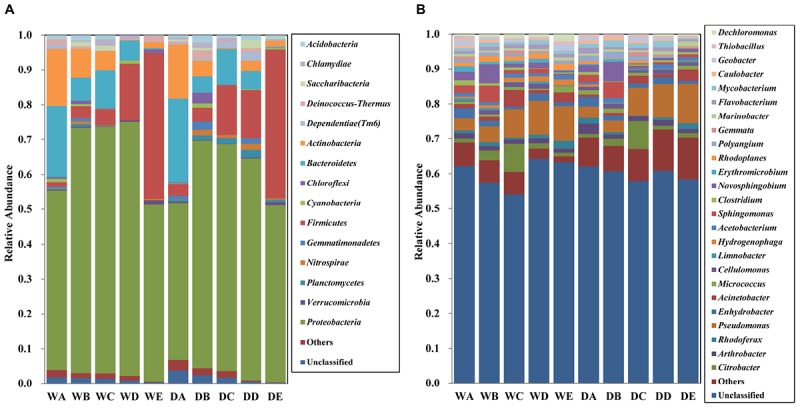
Relative abundances of bacterial community composition at the phylum level **(A)** and genus level **(B)** in all water samples. The rare species with relative abundance less than 0.1% are included as others. A, represents raw water; B, represents quartz sand filtered water; C, represents activated carbon filtered water; D, represents fine filtered water; E, represents final water product, Sample names staring with “W” represents samples collected in the wet season, Sample names staring with “D” represents samples collected in the dry season.

The bacterial community composition of all the water samples was identified at the phylum level (Figure 4A). Overall, 15 bacterial phyla (relative abundance >0.1%) including *Proteobacteria*, *Verrucomicrobia*, *Planctomycetes*, *Nitrospirae*, *Gemmatimonadetes*, *Firmicutes*, *Cyanobacteria*, *Chloroflexi*, *Bacteroidetes*, *Actinobacteria*, *Dependentiae (Tm6)*, *Deinococcus–Thermus*, *Saccharibacteria*, *Chlamydiae*, and *Acidobacteria* were identified in all the water samples. No obvious difference was observed upon comparison of the bacterial microbiota of water samples in different seasons. *Proteobacteria* comprised the dominant bacteria microbiota in all the water samples, accounting for approximately 60% of all the samples, especially in quartz filtered water (69.7%) and activated carbon filtered water (67.5%). The relative abundance of *Proteobacteria* from samples in the wet season was higher than those in the dry season. Along with the treatment processes, significant change in the proportion of *Bacteroidetes*, *Actinobacteria*, and *Firmicutes* was obtained in the MWTS. The relative abundances of *Bacteroidetes* and *Actinobacteria* in raw water were 22.3 and 16.1%, respectively. The proportion of *Bacteroidetes* decreased initially in quartz sand filtered samples, followed by an increase upon activated carbon filtration; however, *Bacteroidetes* from final water product were present a very low levels (<1%) in both seasons. The proportion of *Actinobacteria* gradually decreased in both seasons, being only <1% in final water product. Among all bacterial microbiota, the largest change of relative abundance was observed for *Firmicutes*. The proportion of *Firmicutes* in raw water was 1.4% in the wet season and 3.4% in the dry season, but was highly abundant in finished water in both seasons (41.8 and 42.7%, respectively). The final water product in both seasons mainly comprised *Proteobacteria* (50.8%), *Firmicutes* (42.2%), and *Verrucomicrobia* (1.4%).

The bacterial community composition of all the water samples was identified at the genus level (Figure 4B). Overall, 25 bacterial genera (relative abundance >0.1%), including *Citrobacter*, *Pseudomonas*, *Enhydrobacter*, *Acinetobacter*, *Sphingomonas*, and *Novosphingobium* were detected in all the water samples. The proportion of unclassified genera was range from 60 to 70% in all the samples. The relative abundance of each genus was <3.5% in raw water. Quartz filtered water in both seasons mainly comprised *Novosphingobium* (5.5%) and *Sphingomonas* (4.6%), whereas activated carbon water was dominated by *Pseudomonas* (7.9%), *Citrobacter* (7.8%), and *Acinetobacter* (4.7%). The final water product in both seasons mainly comprised *Pseudomonas* (8.6%), followed by *Acinetobacter* (2.4%).

### Core Microbiome

**FIGURE 5:**
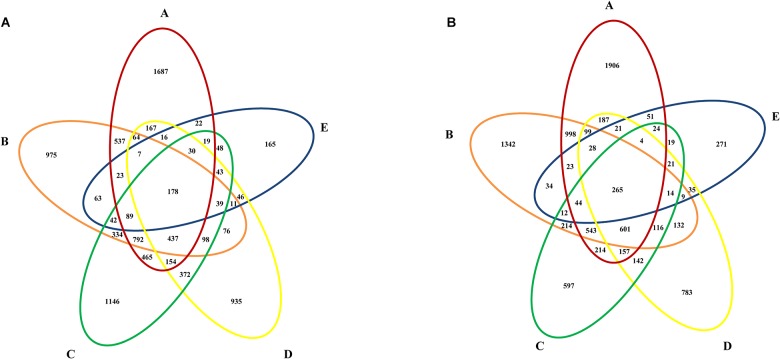
Venn diagrams showing the number of shared OTUs among water samples from different sampling sites during the **(A)** wet season, **(B)** dry season. A, represents raw water; B, represents quartz sand filtered water; C, represents activated carbon filtered water; D, represents fine filtered water; E, represents final water product.

The bacteria that were still present in whole MWTS were defined as the core microbiome (Shade and Handelsman, 2012). Venn diagrams were constructed to investigate the core microbiome in whole MWTS. As shown in Figure 5, 178 (1.9%) OTUs were shared in all the samples from the wet season and 265 (2.9%) from the dry season. Among these shared OTUs, 94 were shared in both seasons and were identified as *Pseudomonadaceae* (*n* = 20), *Sphingomonadaceae* (*n* = 9), *Enterococcaceae* (*n* = 8), unclassified AB024 (*n* = 7), AJ234 (*n* = 6), *Moraxellaceae* (*n* = 6), *Xanthomonadaceae* (*n* = 6), *Comamonadaceae* (*n* = 6), *Citrobacteriaceae* (*n* = 5), *Caulobacteraceae* (*n* = 5), *Bacillaceae* (*n* = 5), *Micrococcaceae* (*n* = 4), *Microbacteriaceae* (*n* = 3), *Mycobacteriaceae* (*n* = 3), and *Nocardiaceae* (*n* = 1). The number of shared OTUs between adjacent treatment steps ranged from 370 to 2127 in samples collected during the wet season and from 397 to 2601 in those collected during the dry season, accounting for 4.1 to 23.4% and 4.4 to 29.2% of overall OTUs, respectively.

## Discussion

In this study, we identified that the microbial activity (Figure 2B) (ATP concentration 5.10 × 10^-11^ – 6.96 × 10^-8^ M) and biomass (Figure 2A) (HPC 2 – 1.97 × 10^3^ CFU/mL) of mineral water samples from an MWTS in the dry season were higher than those (ATP concentration 6.47 × 10^-11^ – 3.32 × 10^-8^ M, HPC 3 – 1.29 × 10^3^ CFU/mL) in the wet season. The seasonal fluctuation of microbial activity and biomass may be attributed to environmental factors (Table 1); specifically, the concentration of TOC. As TOC represents a carbon source, the changing of seasons might alter the nutrient composition of the water environment in the MWTS. The rise of TOC potentially promoting microbial activity and biomass shifts in the dry season in whole MWTS, which is consistent with the description of Li in DWTS (Li et al., 2016). The seasonal fluctuation of microbial activity and biomass had a positive correlation with turbidity and COD. Turbidity is related to suspended, which can adsorb and enrich bacteria. COD is an indicator of organic content and has direct relationship with bacterial biomass. In addition, previous studies in DWTS have shown that environmental parameters such as phosphate, sulfate, pH and water depth were the major factors controlling the temporal variation of bacterial activity and biomass (Lautenschlager et al., 2013; Ji et al., 2015). The temporal pattern of bacteria diversity in DWTS in particular has been thoroughly studied, whereas comparatively little research has been conducted on MWTS. Henne et al. (2013) found obvious seasonal variations of bacterial community composition in surface water reservoirs and Kelly et al. (2014) reported that the diversity of bacteria community in biofilms in the summer months was higher than that observed in the winter. Compared with previous studies in DWTS, the results of the present study showed that the bacterial diversity of water samples have no obvious seasonal variations and bacterial microbiota of water samples in the different season exhibited no obvious difference (Figure 3). The differences in seasonal variations of microbial diversity in the DWTS and MWTS may have been driven by the strong seasonal changes in the DWTS. Previous study also suggested that physicochemical parameters, temperature, and pH, were correlated with bacterial diversity (Chen et al., 2017). In comparison, the stability of the groundwater environment from which the MWTS samples were derived resulted in no obvious seasonal difference of microbial diversity (Fovet et al., 2018). In addition, the groundwater that serves as the raw water of mineral water is not influenced by other external factors as is surface water. For example, after, rainfall or pollution, the surface water bacterial diversity demonstrates significant change (França et al., 2015). In our study, the kinds of bacterial phylum are significantly different from the results of studies in DWTS, which is due to different raw water in DWTS and MWTS.

Distinct differences were identified in the microbial activity, biomass, and diversity at each step of the MWTS. Along with treatment process in whole MWTS, the microbial activity, biomass, and diversity exhibited an obviously downward trend, especially, following the fine filtration and ozone sterilization treatments, which indicated that the water quality was gradually improved. However, after the activated carbon filter treatment process, the activity and diversity of bacteria showed a clear increase in both seasons. Activated carbon filter systems are commonly used for mineral water treatment to ensure equipment life, improve water quality, and prevent pollution (Feng et al., 2013). Activated carbon filters, which contain numerous pores and a large surface area, possess a strong physical adsorption capacity to absorb organic pollutants and microbes (Liao et al., 2013). Our previous study had shown that activated carbon filters could serve as a gathering place for opportunistic pathogens and is the most serious microbial contamination site throughout the whole production process of the MWTS (Wei et al., 2017). The results of the present study thus showed that there was dynamic bacterial community change among the samples of the different treatment processes and that specific treatment processes in the MWTS could affect the bacterial communities in unique ways (Figure 4). In comparison, Poitelon et al. (2010) reported that the influence of water treatment by coagulation-flocculation and sedimentation on bacterial communities is relatively small, whereas Li et al. (2017) observed that bacterial communities significant changed during these steps. Consistent with previous report, the present analysis revealed that *Proteobacteria* predominated in the water samples, which could mainly be related to the capacity of *Proteobacteria* with regard to the biodegradation or biotransformation of various organic compounds (Galvão et al., 2005). In addition, significant change in the proportions of the *Bacteroidetes*, *Actinobacteria*, and *Firmicutes* was also obtained in the MWTS. The activated carbon filtered water was mainly composed of *Proteobacteria* and *Citrobacter*, which differed from the result of DWTS (Lin et al., 2014). Furthermore, cluster analysis indicated that quartz sand filtration apparently changed the bacterial community structure, with *Sphingomonas* and *Novosphingobium* increasing to a higher level in the quartz sand filtered water, which demonstrated that quartz sand filter promoted the proportion of *Sphingomonas* and *Novosphingobium*. However, after ozone sterilization treatment, the proportion of *Firmicutes* exhibited a sharp increase. This result is consistent with the study of Mi et al. (2015) which indicated that disinfection strategies could have a profound impact on the proportion of *Firmicutes* in microbial communities in MWTS. The disinfection resistance mechanism of *Firmicutes* remains unresolved, which may be connected with higher spore resistance. Therefore, additional studies are necessary to validate the effect of disinfection on *Firmicutes* content in mineral water treatment plant bacterial communities.

Despite the spatial-temporal variations of bacterial diversity was detected in MWTS, shared OTUs were found along the treatment processes in different seasons. The bacteria that were present throughout whole MWTS were defined as the core microbiome (Shade and Handelsman, 2012). In the present study, the core microbiome comprised 15 families, which indicated that the core microbiome in MWTS is generally difficult to remove completely and may gradually become a healthy threat (Figure 5). Therefore it is critical to comprehensively understand the core microbiome in MWTS. In particular, some genera, such as *Pseudomonas* and *Acinetobacter*, which are highly likely to contain pathogens and opportunistic pathogens, were retrieved from Illumina amplicons of final water product. The occurrence of these genera in mineral water may increase the risks of waterborne diseases and health problems. For example, some *Acinetobacter* species were important opportunistic pathogens that underlie hospital infections and can cause respiratory tract infection, septicemia, meningitis, endocarditis, and urogenital tract infection (Wong et al., 2017). Some *Mycobacterium* species were the pathogen that causes tuberculosis (Pfyffer, 2015). Among all 300 isolates obtained using cultivation-based techniques in raw water, *Pseudomonas* was predominant species (19.3%). Additionally, Illumina sequencing analysis of 16s rRNA genes showed that the final water product in both seasons mainly comprised *Pseudomonas* (8.6%). *Pseudomonas aeruginosa* serves as an important opportunistic pathogen that is frequently detected in mineral water for human consumption and causes human urinary tract infections (Lu et al., 2016). Our previous research showed that the *Pseudomonas aeruginosa* contamination rate of final water product was 2.3%, which can adversely affect the health of consumers (Wei et al., 2014). Moreover, many waterborne bacteria can form biofilms, which are difficult to remove (Flemming and Wingender, 2010). Although it is possible that PCR amplification might amplify DNA fragment from dead cells, there remains a possibility that some pathogens are able to survive the water treatments considering their resistances to disinfectants. Some pathogen may also cause diseases even at relatively low concentrations (Kirk et al., 2015). Illumina sequencing analysis of 16s rRNA genes in final water product showed that some opportunistic pathogens exist in the final water product that was disinfected by ozone. Hence, manufacturers of mineral water need to established measures for monitoring the MWTS.

This study is the first to investigate the composition and dynamics of bacterial community in a full-scale mineral water treatment plant in China during both the wet and dry seasons. In general, the microbial activity (ATP concentration 5.10 × 10^-11^ – 6.96 × 10^-8^ M) and biomass (HPC 2 – 1.97 × 10^3^ CFU/mL) of mineral water samples from an MWTS in the dry season were higher than those (ATP concentration 6.47 × 10^-11^ – 3.32 × 10^-8^ M, HPC 3 – 1.29 × 10^3^ CFU/mL) in the wet season. All 300 isolates obtained using cultivation-based techniques were distributed in 5 phyla, 7 classes, and 19 genera. *Proteobacteria* accounted for 55.7% (167) of the total isolates and the predominant species comprised *Pseudomonas* (19.3%) at the genus level. Illumina sequencing analysis of 16s rRNA genes revealed that 15 bacterial phyla (relative abundance >0.1%) were detected in all the water samples and that *Proteobacteria* constituted the dominant bacteria microbiota. The bacterial diversity of water samples has no obvious seasonal variations and bacterial microbiota of water samples in the different season exhibited no obvious difference. Distinct differences were identified in the microbial activity, biomass, and diversity at each step of the MWTS. Along with treatment process in whole MWTS, the microbial activity, biomass, and diversity exhibited an obviously downward trend, especially, following the fine filtration and ozone sterilization treatments. In particular, some core microbiome that persisted throughout the treatment process requires special consideration. Notably, opportunistic pathogens including *Pseudomonas*, *Acinetobacter*, *Clostridium*, and *Mycobacterium*, which can adversely affect the health of consumers, were detected throughout the treatment process. These data may therefore provide useful information for the development of public health policies and effective strategies to ensure the safety of mineral water products.

## Author Contributions

QW, JZ, and LW conceived and designed the experiments. LW and WG performed the experiments. LW, QG, and JW analyzed the data. MC, MW, and AL contributed reagents, materials, and analysis tools. LW, HW, and TL contributed to the writing of the manuscript.

## Conflict of Interest Statement

MW and AL were employed by Guangdong Dinghu Mountain Spring Company Limited. The remaining authors declare that the research was conducted in the absence of any commercial or financial relationships that could be construed as a potential conflict of interest.

## Footnotes

1 http://ccb.jhu.edu/software/FLASH/

2 https://mothur.org

3 http://qiime.org/scripts/make_phylogeny.html

4 http://www.canoco5.com/
