# Supplementary material for: Composition and Dynamics of Bacterial Communities in a Full-Scale Mineral Water Treatment Plant
Source: Front Microbiol. 2019 Jul 24;10:1542. doi: 10.3389/fmicb.2019.01542 (PMC6668249; doi:10.3389/fmicb.2019.01542)
Supplement: Supplementary file 1 [file Table_1.docx]

Supplementary Material

# Composition and dynamics of bacterial communities in a full-scale mineral water treatment plant

*Lei Wei^1,2^, Qingping Wu^2*^, Jumei Zhang^2^, Weipeng Guo^2^, Qihui Gu^2^, Huiqing Wu^2^, Juan Wang^3^, Tao Lei^2^, Musheng Wu^4^, Aimei Li^4^*

*** Correspondence:** Qingping Wu: E-mail: [wuqp203@163.com](mailto:wuqp203@163.com)

## Supplementary Table

**Table S1 The information of isolates cultured from raw water sample**

| **Isolates** | **season** | **medium** | **OTU NO.** | **genus** | **phylum** |
| --- | --- | --- | --- | --- | --- |
| SC1 | dry | NA | OUT 1 | *Pseudomonas* | *Proteobacteria* |
| SC2 | dry | NA | OUT 1 | *Pseudomonas* | *Proteobacteria* |
| SC3 | dry | NA | OUT 1 | *Pseudomonas* | *Proteobacteria* |
| SC4 | dry | NA | OUT 17 | *Chloroflexus* | *Chloroflexi* |
| SC5 | dry | NA | OUT 17 | *Chloroflexus* | *Chloroflexi* |
| SC6 | dry | NA | OUT 17 | *Chloroflexus* | *Chloroflexi* |
| SC7 | dry | NA | OUT 8 | *Sphingomonas* | *Proteobacteria* |
| SC8 | dry | NA | OUT 8 | *Sphingomonas* | *Proteobacteria* |
| SC9 | dry | NA | OUT 5 | *Brevundimonas* | *Proteobacteria* |
| SC10 | dry | NA | OUT 5 | *Brevundimonas* | *Proteobacteria* |
| SC11 | dry | NA | OUT 6 | *Caulobacter* | *Proteobacteria* |
| SC12 | dry | NA | OUT 6 | *Caulobacter* | *Proteobacteria* |
| SC13 | dry | NA | OUT 13 | *Arthrobacter* | *Proteobacteria* |
| SC14 | dry | TSA | OUT 1 | *Pseudomonas* | *Proteobacteria* |
| SC15 | dry | TSA | OUT 1 | *Pseudomonas* | *Proteobacteria* |
| SC16 | dry | TSA | OUT 1 | *Pseudomonas* | *Proteobacteria* |
| SC17 | dry | TSA | OUT 1 | *Pseudomonas* | *Proteobacteria* |
| SC18 | dry | TSA | OUT 17 | *Chloroflexus* | *Chloroflexi* |
| SC19 | dry | TSA | OUT 17 | *Chloroflexus* | *Chloroflexi* |
| SC20 | dry | TSA | OUT 8 | *Sphingomonas* | *Proteobacteria* |
| SC21 | dry | TSA | OUT 6 | *Caulobacter* | *Proteobacteria* |
| SC22 | dry | TSA | OUT 6 | *Caulobacter* | *Proteobacteria* |
| SC23 | dry | TSA | OUT 13 | *Arthrobacter* | *Proteobacteria* |
| SC24 | dry | TSA | OUT 13 | *Arthrobacter* | *Proteobacteria* |
| SC25 | dry | TSA | OUT 16 | *Aquimonas* | *Proteobacteria* |
| SC26 | dry | R2A | OUT 1 | *Pseudomonas* | *Proteobacteria* |
| SC27 | dry | R2A | OUT 1 | *Pseudomonas* | *Proteobacteria* |
| SC28 | dry | R2A | OUT 1 | *Pseudomonas* | *Proteobacteria* |
| SC29 | dry | R2A | OUT 17 | *Chloroflexus* | *Chloroflexi* |
| SC30 | dry | R2A | OUT 17 | *Chloroflexus* | *Chloroflexi* |
| SC31 | dry | R2A | OUT 17 | *Chloroflexus* | *Chloroflexi* |
| SC32 | dry | R2A | OUT 8 | *Sphingomonas* | *Proteobacteria* |
| SC33 | dry | R2A | OUT 8 | *Sphingomonas* | *Proteobacteria* |
| SC34 | dry | R2A | OUT 8 | *Sphingomonas* | *Proteobacteria* |
| SC35 | dry | R2A | OUT 5 | *Brevundimonas* | *Proteobacteria* |
| SC36 | dry | R2A | OUT 5 | *Brevundimonas* | *Proteobacteria* |
| SC37 | dry | R2A | OUT 22 | *Microbacterium* | *Actinobacteria* |
| SC38 | dry | R2A | OUT 22 | *Microbacterium* | *Actinobacteria* |
| SC39 | dry | R2A | OUT 3 | *Porphyrobacter* | *Proteobacteria* |
| SC40 | dry | R2A | OUT 3 | *Porphyrobacter* | *Proteobacteria* |
| SC41 | dry | R2A | OUT 21 | *Pimelobacter* | *Actinobacteria* |
| SC42 | dry | R2A | OUT 19 | *Roseomonas* | *Actinobacteria* |
| SC43 | dry | R2A | OUT 9 | *Bacillus* | *Firmicutes* |
| SC44 | dry | R2A | OUT 9 | *Bacillus* | *Firmicutes* |
| SC45 | dry | R2A | OUT 9 | *Bacillus* | *Firmicutes* |
| SC46 | dry | R2A | OUT 2 | *Chryseobacterium* | *Bacteroides* |
| SC47 | dry | R2A | OUT 2 | *Chryseobacterium* | *Bacteroides* |
| SC48 | dry | R2A | OUT 2 | *Chryseobacterium* | *Bacteroides* |
| sbb1 | dry | NA | OUT 1 | *Pseudomonas* | *Proteobacteria* |
| sbb2 | dry | NA | OUT 1 | *Pseudomonas* | *Proteobacteria* |
| sbb3 | dry | NA | OUT 1 | *Pseudomonas* | *Proteobacteria* |
| sbb4 | dry | NA | OUT 16 | *Aquimonas* | *Proteobacteria* |
| sbb5 | dry | NA | OUT 16 | *Aquimonas* | *Proteobacteria* |
| sbb6 | dry | NA | OUT 22 | *Microbacterium* | *Actinobacteria* |
| sbb7 | dry | NA | OUT 22 | *Microbacterium* | *Actinobacteria* |
| sbb8 | dry | NA | OUT 20 | *Chromobacterium* | *Actinobacteria* |
| sbb9 | dry | NA | OUT 20 | *Chromobacterium* | *Actinobacteria* |
| sbb10 | dry | NA | OUT 20 | *Chromobacterium* | *Actinobacteria* |
| sbb11 | dry | NA | OUT 20 | *Chromobacterium* | *Actinobacteria* |
| sbb12 | dry | NA | OUT 20 | *Chromobacterium* | *Actinobacteria* |
| sbb13 | dry | NA | OUT 12 | *Brevibacterium* | *Actinobacteria* |
| sbb14 | dry | NA | OUT 12 | *Brevibacterium* | *Actinobacteria* |
| sbb15 | dry | NA | OUT 12 | *Brevibacterium* | *Actinobacteria* |
| sbb16 | dry | NA | OUT 12 | *Brevibacterium* | *Actinobacteria* |
| sbb17 | dry | NA | OUT 12 | *Brevibacterium* | *Actinobacteria* |
| sbb18 | dry | NA | OUT 7 | *Acinetobacter* | *Proteobacteria* |
| sbb19 | dry | TSA | OUT 1 | *Pseudomonas* | *Proteobacteria* |
| sbb20 | dry | TSA | OUT 1 | *Pseudomonas* | *Proteobacteria* |
| sbb21 | dry | TSA | OUT 1 | *Pseudomonas* | *Proteobacteria* |
| sbb22 | dry | TSA | OUT 1 | *Pseudomonas* | *Proteobacteria* |
| sbb23 | dry | TSA | OUT 8 | *Sphingomonas* | *Proteobacteria* |
| sbb24 | dry | TSA | OUT 8 | *Sphingomonas* | *Proteobacteria* |
| sbb25 | dry | TSA | OUT 5 | *Brevundimonas* | *Proteobacteria* |
| sbb26 | dry | TSA | OUT 5 | *Brevundimonas* | *Proteobacteria* |
| sbb27 | dry | TSA | OUT 22 | *Microbacterium* | *Actinobacteria* |
| sbb28 | dry | TSA | OUT 22 | *Microbacterium* | *Actinobacteria* |
| sbb29 | dry | TSA | OUT 11 | *Acinetobacter* | *Proteobacteria* |
| sbb30 | dry | TSA | OUT 11 | *Acinetobacter* | *Proteobacteria* |
| sbb31 | dry | TSA | OUT 11 | *Acinetobacter* | *Proteobacteria* |
| sbb32 | dry | R2A | OUT 1 | *Pseudomonas* | *Proteobacteria* |
| sbb33 | dry | R2A | OUT 1 | *Pseudomonas* | *Proteobacteria* |
| sbb34 | dry | R2A | OUT 1 | *Pseudomonas* | *Proteobacteria* |
| sbb35 | dry | R2A | OUT 1 | *Pseudomonas* | *Proteobacteria* |
| sbb36 | dry | R2A | OUT 7 | *Acinetobacter* | *Proteobacteria* |
| sbb37 | dry | R2A | OUT 7 | *Acinetobacter* | *Proteobacteria* |
| sbb38 | dry | R2A | OUT 7 | *Acinetobacter* | *Proteobacteria* |
| sbb39 | dry | R2A | OUT 17 | *Chloroflexus* | *Chloroflexi* |
| sbb40 | dry | R2A | OUT 17 | *Chloroflexus* | *Chloroflexi* |
| sbb41 | dry | R2A | OUT 17 | *Chloroflexus* | *Chloroflexi* |
| sbb42 | dry | R2A | OUT 17 | *Chloroflexus* | *Chloroflexi* |
| sbb43 | dry | R2A | OUT 8 | *Sphingomonas* | *Proteobacteria* |
| sbb44 | dry | R2A | OUT 8 | *Sphingomonas* | *Proteobacteria* |
| sbb45 | dry | R2A | OUT 5 | *Brevundimonas* | *Proteobacteria* |
| sbb46 | dry | R2A | OUT 5 | *Brevundimonas* | *Proteobacteria* |
| sbb47 | dry | R2A | OUT 6 | *Caulobacter* | *Proteobacteria* |
| sbb48 | dry | R2A | OUT 6 | *Caulobacter* | *Proteobacteria* |
| sbb49 | dry | R2A | OUT 13 | *Arthrobacter* | *Proteobacteria* |
| sbb50 | dry | R2A | OUT 13 | *Arthrobacter* | *Proteobacteria* |
| sbb51 | dry | R2A | OUT 22 | *Microbacterium* | *Actinobacteria* |
| sbb52 | dry | R2A | OUT 22 | *Microbacterium* | *Actinobacteria* |
| sbb53 | dry | R2A | OUT 9 | *Bacillus* | *Firmicutes* |
| sbb54 | dry | R2A | OUT 9 | *Bacillus* | *Firmicutes* |
| sbb55 | dry | R2A | OUT 9 | *Bacillus* | *Firmicutes* |
| sbb56 | dry | R2A | OUT 3 | *Porphyrobacter* | *Proteobacteria* |
| sbb57 | dry | R2A | OUT 3 | *Porphyrobacter* | *Proteobacteria* |
| sbb58 | dry | R2A | OUT 3 | *Porphyrobacter* | *Proteobacteria* |
| sbb59 | dry | R2A | OUT 9 | *Bacillus* | *Firmicutes* |
| sbb60 | dry | R2A | OUT 9 | *Bacillus* | *Firmicutes* |
| sbb61 | dry | R2A | OUT 9 | *Bacillus* | *Firmicutes* |
| NFKQ1 | dry | NA | OUT 1 | *Pseudomonas* | *Proteobacteria* |
| NFKQ2 | dry | NA | OUT 1 | *Pseudomonas* | *Proteobacteria* |
| NFKQ3 | dry | NA | OUT 1 | *Pseudomonas* | *Proteobacteria* |
| NFKQ4 | dry | NA | OUT 1 | *Pseudomonas* | *Proteobacteria* |
| NFKQ5 | dry | NA | OUT 17 | *Chloroflexus* | *Chloroflexi* |
| NFKQ6 | dry | NA | OUT 17 | *Chloroflexus* | *Chloroflexi* |
| NFKQ7 | dry | NA | OUT 5 | *Brevundimonas* | *Proteobacteria* |
| NFKQ8 | dry | NA | OUT 5 | *Brevundimonas* | *Proteobacteria* |
| NFKQ9 | dry | NA | OUT 6 | *Caulobacter* | *Proteobacteria* |
| NFKQ10 | dry | NA | OUT 6 | *Caulobacter* | *Proteobacteria* |
| NFKQ11 | dry | NA | OUT 16 | *Aquimonas* | *Proteobacteria* |
| NFKQ12 | dry | NA | OUT 16 | *Aquimonas* | *Proteobacteria* |
| NFKQ13 | dry | NA | OUT 20 | *Chromobacterium* | *Actinobacteria* |
| NFKQ14 | dry | NA | OUT 20 | *Chromobacterium* | *Actinobacteria* |
| NFKQ15 | dry | NA | OUT 12 | *Brevibacterium* | *Actinobacteria* |
| NFKQ16 | dry | NA | OUT 12 | *Brevibacterium* | *Actinobacteria* |
| NFKQ17 | dry | NA | OUT 12 | *Brevibacterium* | *Actinobacteria* |
| NFKQ18 | dry | TSA | OUT 1 | *Pseudomonas* | *Proteobacteria* |
| NFKQ19 | dry | TSA | OUT 1 | *Pseudomonas* | *Proteobacteria* |
| NFKQ20 | dry | TSA | OUT 1 | *Pseudomonas* | *Proteobacteria* |
| NFKQ21 | dry | TSA | OUT 8 | *Sphingomonas* | *Proteobacteria* |
| NFKQ22 | dry | TSA | OUT 8 | *Sphingomonas* | *Proteobacteria* |
| NFKQ23 | dry | TSA | OUT 5 | *Brevundimonas* | *Proteobacteria* |
| NFKQ24 | dry | TSA | OUT 5 | *Brevundimonas* | *Proteobacteria* |
| NFKQ25 | dry | TSA | OUT 6 | *Caulobacter* | *Proteobacteria* |
| NFKQ26 | dry | TSA | OUT 6 | *Caulobacter* | *Proteobacteria* |
| NFKQ27 | dry | TSA | OUT 12 | *Brevibacterium* | *Actinobacteria* |
| NFKQ28 | dry | R2A | OUT 1 | *Pseudomonas* | *Proteobacteria* |
| NFKQ29 | dry | R2A | OUT 1 | *Pseudomonas* | *Proteobacteria* |
| NFKQ30 | dry | R2A | OUT 1 | *Pseudomonas* | *Proteobacteria* |
| NFKQ31 | dry | R2A | OUT 1 | *Pseudomonas* | *Proteobacteria* |
| NFKQ32 | dry | R2A | OUT 8 | *Sphingomonas* | *Proteobacteria* |
| NFKQ33 | dry | R2A | OUT 8 | *Sphingomonas* | *Proteobacteria* |
| NFKQ34 | dry | R2A | OUT 20 | *Chromobacterium* | *Actinobacteria* |
| NFKQ35 | dry | R2A | OUT 20 | *Chromobacterium* | *Actinobacteria* |
| NFKQ36 | dry | R2A | OUT 3 | *Porphyrobacter* | *Proteobacteria* |
| NFKQ37 | dry | R2A | OUT 3 | *Porphyrobacter* | *Proteobacteria* |
| NFKQ38 | dry | R2A | OUT 3 | *Porphyrobacter* | *Proteobacteria* |
| NFKQ39 | dry | R2A | OUT 21 | *Pimelobacter* | \| *Actinobacteria* \| \| --- \| |
| NFKQ40 | dry | R2A | OUT 21 | *Pimelobacter* | \| *Actinobacteria* \| \| --- \| |
| NFKQ41 | dry | R2A | OUT 21 | *Pimelobacter* | \| *Actinobacteria* \| \| --- \| |
| NFKQ42 | dry | R2A | OUT 7 | *Acinetobacter* | *Proteobacteria* |
| NFKQ43 | dry | R2A | OUT 7 | *Acinetobacter* | *Proteobacteria* |
| NFKQ44 | dry | R2A | OUT 21 | *Pimelobacter* | \| *Actinobacteria* \| \| --- \| |
| NFKQ45 | dry | R2A | OUT 21 | *Pimelobacter* | \| *Actinobacteria* \| \| --- \| |
| NFKQ46 | dry | R2A | OUT 3 | *Porphyrobacter* | *Proteobacteria* |
| NFKQ47 | dry | R2A | OUT 3 | *Porphyrobacter* | *Proteobacteria* |
| NFKQ48 | dry | R2A | OUT 3 | *Porphyrobacter* | *Proteobacteria* |
| NFKQ49 | dry | R2A | OUT 2 | *Chryseobacterium* | *Bacteroides* |
| NFKQ50 | dry | R2A | OUT 2 | *Chryseobacterium* | *Bacteroides* |
| CSC1 | wet | NA | OUT 1 | *Pseudomonas* | *Proteobacteria* |
| CSC2 | wet | NA | OUT 1 | *Pseudomonas* | *Proteobacteria* |
| CSC3 | wet | NA | OUT 1 | *Pseudomonas* | *Proteobacteria* |
| CSC4 | wet | NA | OUT 8 | *Sphingomonas* | *Proteobacteria* |
| CSC5 | wet | NA | OUT 8 | *Sphingomonas* | *Proteobacteria* |
| CSC6 | wet | NA | OUT 16 | *Aquimonas* | *Proteobacteria* |
| CSC7 | wet | NA | OUT 16 | *Aquimonas* | *Proteobacteria* |
| CSC8 | wet | NA | OUT 22 | *Microbacterium* | *Actinobacteria* |
| CSC9 | wet | NA | OUT 22 | *Microbacterium* | *Actinobacteria* |
| CSC10 | wet | NA | OUT 20 | *Chromobacterium* | *Actinobacteria* |
| CSC11 | wet | NA | OUT 20 | *Chromobacterium* | *Actinobacteria* |
| CSC12 | wet | NA | OUT 15 | *Chryseobacterium* | *Bacteroides* |
| CSC13 | wet | NA | OUT 15 | *Chryseobacterium* | *Bacteroides* |
| CSC14 | wet | TSA | OUT 5 | *Brevundimonas* | *Proteobacteria* |
| CSC15 | wet | TSA | OUT 5 | *Brevundimonas* | *Proteobacteria* |
| CSC16 | wet | TSA | OUT 6 | *Caulobacter* | *Proteobacteria* |
| CSC17 | wet | TSA | OUT 6 | *Caulobacter* | *Proteobacteria* |
| CSC18 | wet | TSA | OUT 13 | *Arthrobacter* | *Proteobacteria* |
| CSC19 | wet | TSA | OUT 13 | *Arthrobacter* | *Proteobacteria* |
| CSC20 | wet | TSA | OUT 16 | *Aquimonas* | *Proteobacteria* |
| CSC21 | wet | TSA | OUT 16 | *Aquimonas* | *Proteobacteria* |
| CSC22 | wet | TSA | OUT 4 | *Xanthomonas* | *Bacteroides* |
| CSC23 | wet | TSA | OUT 4 | *Xanthomonas* | *Bacteroides* |
| CSC24 | wet | TSA | OUT 4 | *Xanthomonas* | *Bacteroides* |
| CSC25 | wet | R2A | OUT 1 | *Pseudomonas* | *Proteobacteria* |
| CSC26 | wet | R2A | OUT 1 | *Pseudomonas* | *Proteobacteria* |
| CSC27 | wet | R2A | OUT 1 | *Pseudomonas* | *Proteobacteria* |
| CSC28 | wet | R2A | OUT 1 | *Pseudomonas* | *Proteobacteria* |
| CSC29 | wet | R2A | OUT 17 | *Chloroflexus* | *Chloroflexi* |
| CSC30 | wet | R2A | OUT 17 | *Chloroflexus* | *Chloroflexi* |
| CSC31 | wet | R2A | OUT 8 | *Sphingomonas* | *Proteobacteria* |
| CSC32 | wet | R2A | OUT 8 | *Sphingomonas* | *Proteobacteria* |
| CSC33 | wet | R2A | OUT 5 | *Brevundimonas* | *Proteobacteria* |
| CSC34 | wet | R2A | OUT 5 | *Brevundimonas* | *Proteobacteria* |
| CSC35 | wet | R2A | OUT 20 | *Chromobacterium* | *Actinobacteria* |
| CSC36 | wet | R2A | OUT 20 | *Chromobacterium* | *Actinobacteria* |
| CSC37 | wet | R2A | OUT 20 | *Chromobacterium* | *Actinobacteria* |
| CSC38 | wet | R2A | OUT 12 | *Brevibacterium* | *Actinobacteria* |
| CSC39 | wet | R2A | OUT 12 | *Brevibacterium* | *Actinobacteria* |
| CSC40 | wet | R2A | OUT 18 | *Mycobacterium* | *Actinobacteria* |
| CSC41 | wet | R2A | OUT 18 | *Mycobacterium* | *Actinobacteria* |
| CSC42 | wet | R2A | OUT 18 | *Mycobacterium* | *Actinobacteria* |
| CSC43 | wet | R2A | OUT 7 | *Acinetobacter* | *Proteobacteria* |
| CSC44 | wet | R2A | OUT 7 | *Acinetobacter* | *Proteobacteria* |
| CSC45 | wet | R2A | OUT 2 | *Chryseobacterium* | *Bacteroides* |
| CSC46 | wet | R2A | OUT 2 | *Chryseobacterium* | *Bacteroides* |
| CSC47 | wet | R2A | OUT 9 | *Bacillus* | *Firmicutes* |
| CSC48 | wet | R2A | OUT 9 | *Bacillus* | *Firmicutes* |
| GZJT1 | wet | NA | OUT 10 | *Pseudomonas* | *Proteobacteria* |
| GZJT2 | wet | NA | OUT 10 | *Pseudomonas* | *Proteobacteria* |
| GZJT3 | wet | NA | OUT 10 | *Pseudomonas* | *Proteobacteria* |
| GZJT4 | wet | NA | OUT 17 | *Chloroflexus* | *Chloroflexi* |
| GZJT5 | wet | NA | OUT 17 | *Chloroflexus* | *Chloroflexi* |
| GZJT6 | wet | NA | OUT 13 | *Arthrobacter* | *Proteobacteria* |
| GZJT7 | wet | NA | OUT 16 | *Aquimonas* | *Proteobacteria* |
| GZJT8 | wet | NA | OUT 16 | *Aquimonas* | *Proteobacteria* |
| GZJT9 | wet | NA | OUT 22 | *Microbacterium* | *Actinobacteria* |
| GZJT10 | wet | NA | OUT 22 | *Microbacterium* | *Actinobacteria* |
| GZJT11 | wet | NA | OUT 12 | *Brevibacterium* | *Actinobacteria* |
| GZJT12 | wet | NA | OUT 12 | *Brevibacterium* | *Actinobacteria* |
| GZJT13 | wet | NA | OUT 12 | *Brevibacterium* | *Actinobacteria* |
| GZJT14 | wet | NA | OUT 12 | *Brevibacterium* | *Actinobacteria* |
| GZJT15 | wet | TSA | OUT 12 | *Brevibacterium* | *Actinobacteria* |
| GZJT16 | wet | TSA | OUT 8 | *Sphingomonas* | *Proteobacteria* |
| GZJT17 | wet | TSA | OUT 13 | *Arthrobacter* | *Proteobacteria* |
| GZJT18 | wet | TSA | OUT 13 | *Arthrobacter* | *Proteobacteria* |
| GZJT19 | wet | TSA | OUT 22 | *Microbacterium* | *Actinobacteria* |
| GZJT20 | wet | TSA | OUT 22 | *Microbacterium* | *Actinobacteria* |
| GZJT21 | wet | TSA | OUT 12 | *Brevibacterium* | *Actinobacteria* |
| GZJT22 | wet | TSA | OUT 12 | *Brevibacterium* | *Actinobacteria* |
| GZJT23 | wet | TSA | OUT 12 | *Brevibacterium* | *Actinobacteria* |
| GZJT24 | wet | TSA | OUT 12 | *Brevibacterium* | *Actinobacteria* |
| GZJT25 | wet | R2A | OUT 10 | *Pseudomonas* | *Proteobacteria* |
| GZJT26 | wet | R2A | OUT 10 | *Pseudomonas* | *Proteobacteria* |
| GZJT27 | wet | R2A | OUT 10 | *Pseudomonas* | *Proteobacteria* |
| GZJT28 | wet | R2A | OUT 10 | *Pseudomonas* | *Proteobacteria* |
| GZJT29 | wet | R2A | OUT 17 | *Chloroflexus* | *Chloroflexi* |
| GZJT30 | wet | R2A | OUT 17 | *Chloroflexus* | *Chloroflexi* |
| GZJT31 | wet | R2A | OUT 23 | *Hydrogenophaga* | *Proteobacteria* |
| GZJT32 | wet | R2A | OUT 23 | *Hydrogenophaga* | *Proteobacteria* |
| GZJT33 | wet | R2A | OUT 4 | *Xanthomonas* | *Bacteroides* |
| GZJT34 | wet | R2A | OUT 4 | *Xanthomonas* | *Bacteroides* |
| GZJT35 | wet | R2A | OUT 4 | *Xanthomonas* | *Bacteroides* |
| GZJT36 | wet | R2A | OUT 19 | *Roseomonas* | *Actinobacteria* |
| GZJT37 | wet | R2A | OUT 19 | *Roseomonas* | *Actinobacteria* |
| GZJT38 | wet | R2A | OUT 9 | *Bacillus* | *Firmicutes* |
| GZJT39 | wet | R2A | OUT 9 | *Bacillus* | *Firmicutes* |
| GZJT40 | wet | R2A | OUT 9 | *Bacillus* | *Firmicutes* |
| BM1 | wet | NA | OUT 10 | *Pseudomonas* | *Proteobacteria* |
| BM2 | wet | NA | OUT 10 | *Pseudomonas* | *Proteobacteria* |
| BM3 | wet | NA | OUT 10 | *Pseudomonas* | *Proteobacteria* |
| BM4 | wet | NA | OUT 10 | *Pseudomonas* | *Proteobacteria* |
| BM5 | wet | NA | OUT 17 | *Chloroflexus* | *Chloroflexi* |
| BM6 | wet | NA | OUT 17 | *Chloroflexus* | *Chloroflexi* |
| BM7 | wet | NA | OUT 5 | *Brevundimonas* | *Proteobacteria* |
| BM8 | wet | NA | OUT 5 | *Brevundimonas* | *Proteobacteria* |
| BM9 | wet | NA | OUT 16 | *Aquimonas* | *Proteobacteria* |
| BM10 | wet | NA | OUT 16 | *Aquimonas* | *Proteobacteria* |
| BM11 | wet | NA | OUT 16 | *Aquimonas* | *Proteobacteria* |
| BM12 | wet | NA | OUT 22 | *Microbacterium* | *Actinobacteria* |
| BM13 | wet | NA | OUT 22 | *Microbacterium* | *Actinobacteria* |
| BM14 | wet | NA | OUT 14 | *Xanthomonas* | *Bacteroides* |
| BM15 | wet | NA | OUT 14 | *Xanthomonas* | *Bacteroides* |
| BM16 | wet | NA | OUT 14 | *Xanthomonas* | *Bacteroides* |
| BM17 | wet | NA | OUT 14 | *Xanthomonas* | *Bacteroides* |
| BM18 | wet | NA | OUT 2 | *Chryseobacterium* | *Bacteroides* |
| BM19 | wet | TSA | OUT 10 | *Pseudomonas* | *Proteobacteria* |
| BM20 | wet | TSA | OUT 10 | *Pseudomonas* | *Proteobacteria* |
| BM21 | wet | TSA | OUT 10 | *Pseudomonas* | *Proteobacteria* |
| BM22 | wet | TSA | OUT 8 | *Sphingomonas* | *Proteobacteria* |
| BM23 | wet | TSA | OUT 8 | *Sphingomonas* | *Proteobacteria* |
| BM24 | wet | TSA | OUT 6 | *Caulobacter* | *Proteobacteria* |
| BM25 | wet | TSA | OUT 6 | *Caulobacter* | *Proteobacteria* |
| BM26 | wet | TSA | OUT 22 | *Microbacterium* | *Actinobacteria* |
| BM27 | wet | TSA | OUT 22 | *Microbacterium* | *Actinobacteria* |
| BM28 | wet | TSA | OUT 14 | *Xanthomonas* | *Bacteroides* |
| BM29 | wet | TSA | OUT 14 | *Xanthomonas* | *Bacteroides* |
| BM30 | wet | TSA | OUT 10 | *Pseudomonas* | *Proteobacteria* |
| BM31 | wet | R2A | OUT 10 | *Pseudomonas* | *Proteobacteria* |
| BM32 | wet | R2A | OUT 10 | *Pseudomonas* | *Proteobacteria* |
| BM33 | wet | R2A | OUT 10 | *Pseudomonas* | *Proteobacteria* |
| BM34 | wet | R2A | OUT 10 | *Pseudomonas* | *Proteobacteria* |
| BM35 | wet | R2A | OUT 17 | *Chloroflexus* | *Chloroflexi* |
| BM36 | wet | R2A | OUT 17 | *Chloroflexus* | *Chloroflexi* |
| BM37 | wet | R2A | OUT 8 | *Sphingomonas* | *Proteobacteria* |
| BM38 | wet | R2A | OUT 8 | *Sphingomonas* | *Proteobacteria* |
| BM39 | wet | R2A | OUT 5 | *Brevundimonas* | *Proteobacteria* |
| BM40 | wet | R2A | OUT 5 | *Brevundimonas* | *Proteobacteria* |
| BM41 | wet | R2A | OUT 6 | *Caulobacter* | *Proteobacteria* |
| BM42 | wet | R2A | OUT 6 | *Caulobacter* | *Proteobacteria* |
| BM43 | wet | R2A | OUT 23 | *Hydrogenophaga* | *Proteobacteria* |
| BM44 | wet | R2A | OUT 23 | *Hydrogenophaga* | *Proteobacteria* |
| BM45 | wet | R2A | OUT 18 | *Mycobacterium* | *Actinobacteria* |
| BM46 | wet | R2A | OUT 18 | *Mycobacterium* | *Actinobacteria* |
| BM47 | wet | R2A | OUT 18 | *Mycobacterium* | *Actinobacteria* |
| BM48 | wet | R2A | OUT 19 | *Roseomonas* | *Actinobacteria* |
| BM49 | wet | R2A | OUT 19 | *Roseomonas* | *Actinobacteria* |
| BM50 | wet | R2A | OUT 15 | *Chryseobacterium* | *Bacteroides* |
| BM51 | wet | R2A | OUT 15 | *Chryseobacterium* | *Bacteroides* |
| BM52 | wet | R2A | OUT 19 | *Roseomonas* | *Actinobacteria* |
| BM53 | wet | R2A | OUT 19 | *Roseomonas* | *Actinobacteria* |
